# Identification of 5-Hydroxymethylfurfural (5-HMF) as an Active Component Citrus Jabara That Suppresses FcεRI-Mediated Mast Cell Activation

**DOI:** 10.3390/ijms21072472

**Published:** 2020-04-02

**Authors:** Ryota Uchida, Michiko Kato, Yuka Hattori, Hiroko Kikuchi, Emi Watanabe, Katsuumi Kobayashi, Keigo Nishida

**Affiliations:** 1 Laboratory of Immune Regulation, Graduate School of Pharmaceutical Sciences, Suzuka University of Medical Science, 3500-3 Minamitamagaki, Suzuka Mie 513-8607, Japan; mumyojoyani@gmail.com (R.U.); m-kato@suzuka-u.ac.jp (M.K.); 2 Laboratory of Immune Regulation, Faculty of Pharmaceutical Sciences, Suzuka University of Medical Science, 3500-3 Minamitamagaki-cho, Suzuka Mie 513-8670, Japan; 3 Development Planning Department, Nisshin Honey Co.,LTD, 3133-1 Maki Anpachi-cho, Anpachi-gun, Gifu 503-0125, Japan; hiroko.kikuchi@nisshin-honey.co.jp (H.K.); emi.watanabe@nisshin-honey.co.jp (E.W.); katsuumi.kobayashi@nisshin-honey.co.jp (K.K.)

**Keywords:** mast cells, allergy, degranulation, cytokine, *Citrus jabara*, fermentation, 5-HMF

## Abstract

Jabara (*Citrus jabara* Hort. ex Y. Tanaka) is a type of citrus fruit known for its beneficial effect against seasonal allergies. Jabara is rich in the antioxidant narirutin whose anti-allergy effect has been demonstrated. One of the disadvantages in consuming Jabara is its bitter flavor. Therefore, we fermented the fruit to reduce the bitterness and make Jabara easy to consume. Here, we examined whether fermentation alters the anti-allergic property of Jabara. Suppression of degranulation and cytokine production was observed in mast cells treated with fermented Jabara and the effect was dependent on the length of fermentation. We also showed that 5-hydroxymethylfurfural (5-HMF) increases as fermentation progresses and was identified as an active component of fermented Jabara, which inhibited mast cell degranulation. Mast cells treated with 5-HMF also exhibited reduced degranulation and cytokine production. In addition, we showed that the expression levels of phospho-PLC**γ**1 and phospho-ERK1/2 were markedly reduced upon FcεRI stimulation. These results indicate that 5-HMF is one of the active components of fermented Jabara that is involved in the inhibition of mast cell activation.

## 1. Introduction

Millions of people who react to airborne allergens such as pollens suffer from symptoms including rhinitis, atopic dermatitis, and asthma, which could reduce the patients’ quality of life. These allergic symptoms are induced by the activation of inflammatory cells such as mast cells [1,2,3].

Mast cells express Fc epsilon receptor (FcεRI), which can bind to immunoglobulin E (IgE). Stimulation of IgE-sensitized mast cells with a specific antigen results in a cascade of events leading to the secretion and production of proinflammatory molecules such as histamine, lipid mediators and cytokines [4,5]. These secreted molecules play critical roles in the inflammatory reactions in patients with allergic diseases. Allergic symptoms can impair the patients’ quality of life by causing chronic fatigue, cognitive impairment, and many other symptoms associated with the condition. While there are many anti-allergy medications on the market, more effective and safer drugs are still being sought. Use of natural products for the prevention and treatment of allergic diseases is an attractive and advantageous option in that they have fewer side effects.

*Citrus jabara* is a cultivar of citrus native to Kitayama village in Wakayama prefecture, Japan. Jabara has gained much attention in recent years for its effect on alleviating seasonal allergy symptoms [6]. Jabara has been found to be particularly rich in the dietary flavonoid narirutin (naringenin-7-*O*-β-D-rutinoside) commonly found in citrus that is reported to possess anti-allergic property. The anti-allergic property of narirutin has been shown in both in vitro and in vivo studies using a mouse model of atopic dermatitis and airway inflammation [7,8,9]. However, it is still unknown whether Jabara contains other active compounds that have anti-allergic activity.

While the consumption of Jabara has been shown to be effective in alleviating allergic symptoms in patients suffering from seasonal allergy, its use as a health food has some restrictions due to its flavor, especially the bitterness of the peel where narirutin is primarily present. To overcome this disadvantage, we reduced the bitterness of the fruit by processing it with our proprietary procedure that incorporates self-fermentation and maturation. However, it is unknown whether Jabara’s chemical composition changes and its anti-allergic effects are affected by fermentation.

In this study, we investigated the effect of fermented Jabara extract on allergic responses by examining in vitro anti-allergic activity using mouse mast cells. We identified 5-hydroxymethylfurfural (5-HMF) as an active compound that inhibits FcεRI-mediated degranulation and cytokine production in mouse mast cells.

## 2. Results

### 2.1. Fermented Jabara Suppressed Mast Cell Activation

**Figure 1:**
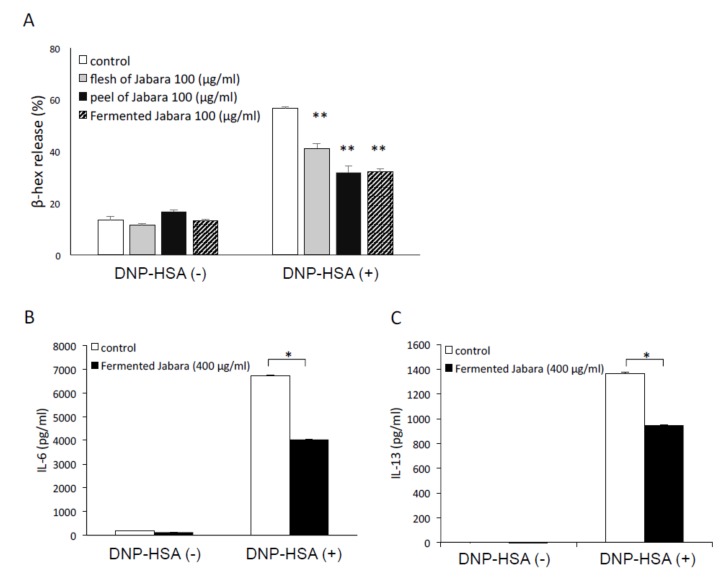
(**A**) Inhibitory effect of DMSO extract of flesh and peel of raw Jabara, as well as the extract of fermented Jabara (four weeks) on β-hexosaminidase (β-hex) release from mast cells. IgE-sensitized mast cells (1.0 × 10^5^ cells/well) were preincubated with each extract at 37 °C for 15 min prior to 30 min stimulation with DNP-HSA. Data represent one of at least three trials that showed similar results. Data are presented as mean (SD). **p < 0.01, two-tailed Student’s *t*-test. (**B**,**C**) Inhibitory effect of DMSO extract of fermented Jabara on cytokine production in mast cells. Mast cells were stimulated with 10 ng/mL DNP-HSA for 6 h with or without the addition of fermented Jabara. IL-6 (B) and IL-13 (C) production were measured by ELISA. Data represent one of at least three trials that showed similar results. Data are presented as mean (SD). *p < 0.05, two-tailed Student’s *t*-test.

We first examined the effect of fermented Jabara on the activation of mast cells by measuring the release of the mast cell degranulation marker β-hexosaminidase. Antigen-dependent degranulation of mast cells was significantly suppressed by pretreating the cells with four-week fermented Jabara as well as flesh and peel of raw Jabara (Figure 1A). Next, we measured the concentration of cytokines in the culture supernatant of mast cells by ELISA (Figure 1B,C). Cytokine production was suppressed by pretreating mast cells with fermented Jabara. In addition, no significant change in the cell viability was observed after 6-h exposure to Jabara extracts (Figure S1). These data suggest that inhibition of mast cell degranulation and cytokine production induced by antigen stimulation is not due to cytotoxic effect of Jabara extracts.

**Figure 2:**
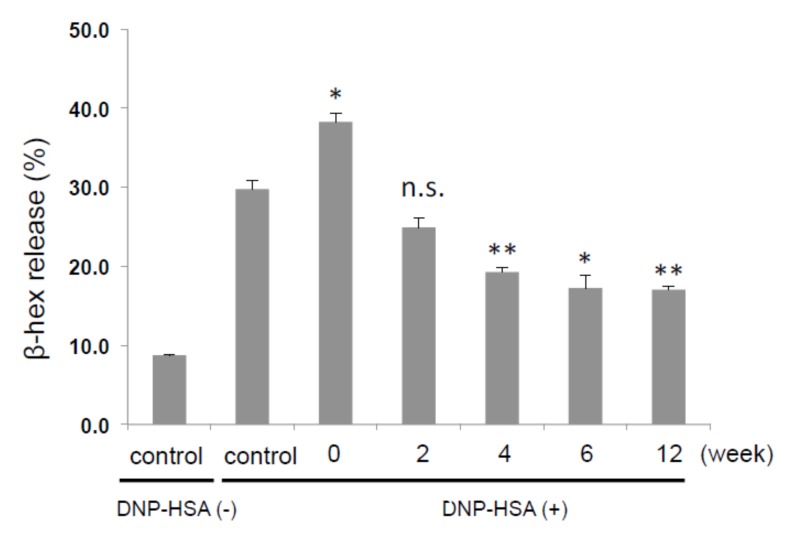
Inhibitory effect of DMSO extract from fermented Jabara on β-hex release from mast cells. IgE-sensitized mast cells (1.0 × 10^5^ cells/well) were preincubated with each extract (200 μg/mL) at 37 °C for 15 min prior to 30 min stimulation with DNP-HSA. Results represent one trial. At least three additional trials show similar results. Data are presented as mean + SD. *p < 0.05, **p < 0.01, two-tailed Student’s **t**-test. n.s., not significant.

We then examined whether the length of fermentation influences the effectiveness of inhibition of degranulation. As shown in Figure 2, the inhibitory effect of Jabara increased as the length of fermentation increased. Degranulation was significantly inhibited when cells were treated with Jabara fermented for 12 weeks.

### 2.2. 5-HMF is the Active Component in Fermented Jabara that Inhibits Mast Cell Activation

**Figure 3:**
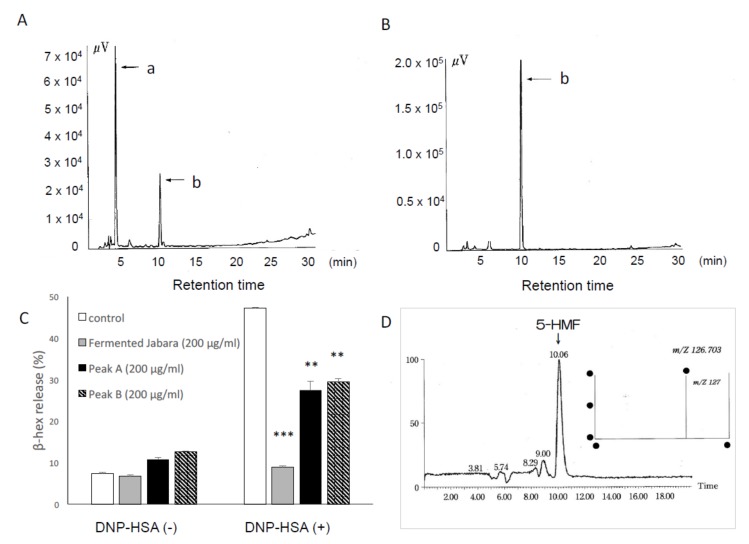
HPLC chromatograms of extracts (DMSO: MeOH = 1:1) of fermented Jabara (**A**) and flesh of Jabara (**B**). Column: ODS-HG-5 (φ4.6 × 250 mm, 5 μm) NOMURA CHEMICAL; flow rate, 1.0 mL/min; detected with UV at 285 nm. (**C**) Inhibitory effect of the compounds in peak *a* and *b* extracted from fermented Jabara on β-hex release from mast cells. IgE-sensitized mast cells (1.0 × 10^5^ cells/well) were preincubated with each extract at 37 °C for 15 min prior to 30 min stimulation with DNP-HSA. Data represent one of at least three trials that showed similar results. Data are presented as mean (SD). **p < 0.01, ***p < 0.001, one-way ANOVA with Tukey–Kramer multiple comparison test. (**D**) LC-MS analysis of peak *a* revealed its molecular weight.

To identify the active component of fermented Jabara that exerts inhibitory effect against mast cell degranulation, we fractionated the extract of fermented Jabara by HPLC and examined the inhibitory effect of each fraction on degranulation. The chromatogram of the fermented sample showed two major peaks at 3.7 min (peak *a*) and 9.85 min (peak *b*) (Figure 3A). The retention time of peak *b* in the fermented sample as well as the single peak in the unfermented sample corresponded to the retention time of the narirutin standard (data not shown). The 3.7 min peak appeared in the fermented sample (Figure 3A) but not in the unfermented sample (Figure 3B), suggesting that this compound is produced by fermentation. In addition, the amount of this compound increased as the fermentation progressed.

We then analyzed the fraction containing the compound in peak *a* for its effect on mast cell degranulation. As shown in Figure 3C, the compound in peak *a* exerted similar inhibitory effect on degranulation as the compound in peak *b* (Narirutin).

Peak *a* was identified as 5-hydroxymethylfurfural (5-HMF) on the basis of ^1^H NMR, ^13^C NMR LC-MS, and HPLC. The ^13^C NMR spectrum of the compound recovered from peak *a* revealed six signals, indicating the presence of six (or a multiple of six) carbon atoms. When these signals were compared to the reference signals from the Aldrich Spectral Library, they corresponded with the NMR profile of 5-HMF This compound was further analyzed by LC-MS, and the MS spectrum showed the ion peak at *m/z* 126, which matched that of 5-HMF (Figure 3D, right). The compound from peak *a* also eluted at the same retention time as the 5-HMF standard. Based on these analyses, we identified this compound as 5-HMF.

### 2.3. 5-HMF Increased as Fermentation Progressed

**Figure 4:**
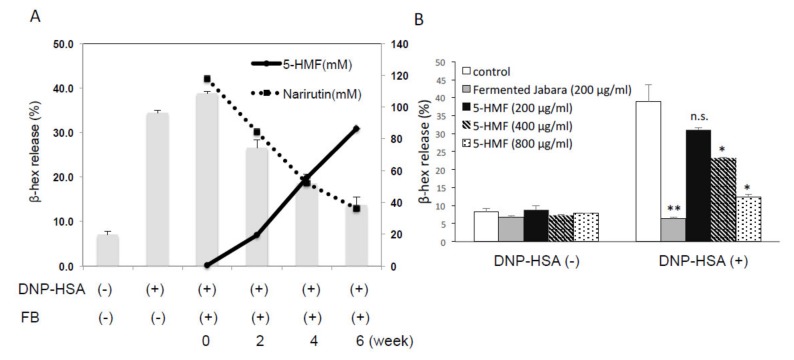
(**A**) Inhibitory effect of the DMSO extract of fermented Jabara (FB) on β-hex release from mast cells. IgE-sensitized mast cells (1.0 × 10^5^ cells / well) were preincubated with each extract (200 ug/mL) at 37 °C for 15 min prior to 30 min stimulation with DNP-HSA. Data are presented as mean (SD). Solid and dashed lines: Amount of 5-HMF (mM) and Narirutin (mM) in Jabara, respectively, determined on different days of fermentation by HPLC analysis. (**B**) Inhibitory effect of 5-HMF on β-hex release from mast cells. IgE-sensitized mast cells (1.0 × 10^5^ cells/well) were preincubated with each extract at 37 °C for 15 min prior to 30 min stimulation with DNP-HSA. Results represent one trial. At least three additional trials show similar results. Data are presented as mean (SD). *p < 0.05, **p < 0.01, two-tailed Student’s *t*-test. n.s., not significant.

HPLC analysis showed that unfermented Jabara is rich in narirutin while 5-HMF was barely detectable. As fermentation progressed, the amount of 5-HMF increased with a concomitant decrease of narirutin. After 4–6 weeks of fermentation, the amount of 5-HMF equaled or exceeded the amount of narirutin (Figure 4A).

The effect of 5-HMF on degranulation was examined using various concentrations of 5-HMF standard and Jabara fermented for four weeks. As shown in Figure 4B, β-hexosaminidase release was suppressed by 5-HMF in a concentration-dependent manner. Fermented Jabara suppressed β-hexosaminidase release to the basal level, suggesting that the anti-degranulation effect of Jabara becomes more potent as fermentation time increases. In addition, no significant change in the cell viability was observed after 6-h exposure to various doses of 5-HMF (Figure S1). These data suggested that inhibition of mast cell degranulation induced by antigen stimulation is not due to cytotoxic effect of 5-HMF.

### 2.4. Effect of 5-HMF on the Expression of Proinflammatory Cytokine

**Figure 5:**
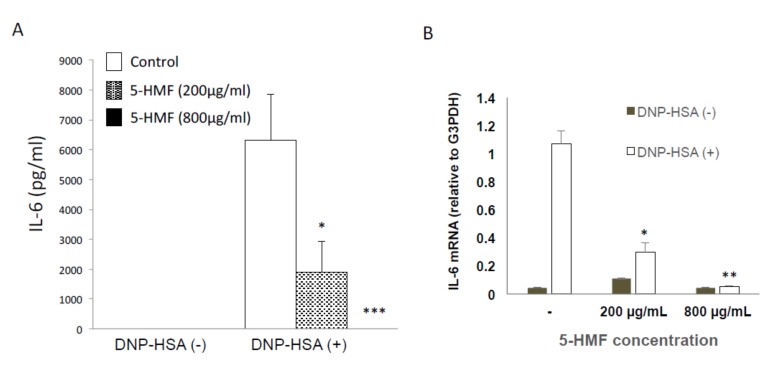
Inhibitory effect of 5-HMF on cytokine production and gene induction in mast cells. (**A**) Mast cells were stimulated with 10 ng/mL DNP-HSA for 6 h and 5-HMF was added at the time of stimulation. IL-6 production was then measured by ELISA. Data are from a representative of at least three trials that showed similar results. Data are presented as mean (SD). *p < 0.05, ***p < 0.001, two-tailed Student’s *t*-test. (**B**) Expression of IL-6 mRNA relative to G3PDH mRNA. Mast cells were stimulated with 10 ng/mL DNP-HSA for 1 h in the presence of 200 or 800 μg/mL 5-HMF. A representative of three experiments is shown. Error bars represent standard deviation among duplicate samples. *p < 0.05, **p < 0.01, two-tailed Student’s *t*-test.

Next, we examined whether 5-HMF inhibits cytokine production. The effect of 5-HMF on the expression of the proinflammatory cytokine IL-6 in FcεRI-induced mast cells was assessed by ELISA. When cells were pretreated with 5-HMF, the expression level of IL-6 was significantly decreased in a concentration-dependent manner as compared with the untreated group (Figure 5). Next, we analyzed the mRNA levels of cytokines by quantitative PCR. Antigen stimulation of mast cells led to an increase in the *Il6* mRNA level (Figure 5B), and treating these cells with 5-HMF decreased the *Il6* mRNA level in a dose-dependent manner (Figure 5B). These results indicate that 5-HMF is capable of suppressing the induction and production of proinflammatory cytokines.

### 2.5. The effect of 5-HMF on the PLCγ and MAPK Signaling Pathways

**Figure 6:**
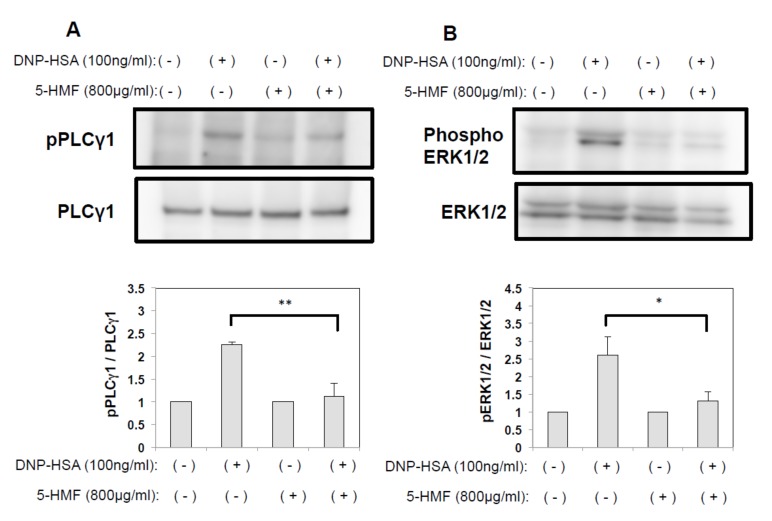
(**A**) Inhibition of PLCγ1 phosphorylation in mast cells treated with 5-HMF. After stimulation with DNP-HSA, mast cells were lysed and the cytosol fraction was immunoblotted with anti-phospho-PLCγ1 (Top). Data were normalized to the expression levels of non-phosphorylated PLCγ1 (Bottom). (**B**) Inhibition ERK1/2 phosphorylation in mast cells treated with 5-HMF. After stimulation with DNP-HSA, mast cells were lysed and the cytosol fraction was immunoblotted with anti-phospho-ERK1/2 (Top). Data were normalized to the expression levels of non-phosphorylated ERK1/2 (Bottom). Data are presented as mean (SD). *p < 0.05, **p < 0.01, two-tailed Student’s *t*-test.

To gain some insight into the molecular mechanisms involved in the suppression on FcεRI-stimulated mast cell activation by 5-HMF, we examined the MAPK signaling pathway, which is known to regulate the expression of proinflammatory genes in response to FcεRI stimulation [10]. We hypothesized that 5-HMF downregulates cytokine expression by suppressing the activation of MAPK signaling, and analyzed the phosphorylation status of PLC**γ** and ERK1/2 by Western blot analysis. The expression levels of unphosphorylated PLC**γ**1 and ERK1/2 were comparable among all treatment groups. In addition, phosphorylated proteins were marginally expressed in the negative control group (no 5-HMF pretreatment, no stimulation) but were highly expressed in cells stimulated with DNP-HSA. Phosphorylation of both PLCγ1 and ERK1/2 was completely suppressed by treating mast cells with 800 µg/mL 5-HMF (Figure 6). Thus, our results support the idea that 5-HMF inhibits the expression of cytokine expression by suppressing the MAPK signaling pathways.

## 3. Discussion

This study examined the anti-allergic effect of citrus Jabara that was fermented in order to reduce bitterness. We showed that β-hexosaminidase release was significantly decreased in mast cells treated with fermented Jabara, and the effect was enhanced as the fermentation period increased.

Currently, the flavonoid narirutin is believed to be the major contributor of anti-allergic effect in Jabara. It has been shown that narirutin inhibits histamine release from rat peritoneal mast cells in vitro and exerts an inhibitory effect on allergic skin reactions in a murine model of atopic dermatitis [8]. Interestingly, the amount of narirutin abundant in raw Jabara fruit decreased as fermentation progressed, while the amount of 5-HMF, which is barely present in raw fruit, increased. We also observed that the extract of freeze-dried unfermented Jabara, which is expected to be rich in narirutin (Figure 2), did not inhibit mast cell degranulation. Since the HPLC-purified compound *b* (narirutin) suppressed degranulation (Figure 3C), we considered the possibility that there is an unidentified factor in the freeze-dried peel that interferes with the effect of narirutin.

Our data indicate that the fermentation process, during which narirutin is decreased, does not affect the anti-allergic effect of Jabara. We also observed that the amount of 5-HMF and the anti-degranulation effect increased proportionately to the length of fermentation, which led us to further investigate 5-HMF as the major anti-allergic/anti-inflammatory agent in fermented Jabara.

5-HMF is an organic compound generated by sugar reduction through the Maillard reaction [11,12,13]; therefore, it is normally present in heat-processed foods such as honey, dried fruits, and fruit juices [14,15,16]. The anti-inflammatory and anti-allergic effects of 5-HMF have been reported in several recent studies [17,18,19,20,21]. For example, it has been reported that *Lycium chinense*, commonly called wolfberry, contains large amount of 5-HMF and that the fruit extract inhibits degranulation of rat basophilic leukemia (RBL) cells [17]. In addition, Kong et al. showed that lipopolysaccharide (LPS)-stimulated production of pro-inflammatory cytokines was suppressed in RAW 264.7 cells pretreated with 5-HMF [21].

With regards to the mechanism underlying the anti-inflammatory activities of 5-HMF, little has been understood. Yamada et al. described that 5-HMF inhibits the release of chemical mediators by suppressing Ca^2+^ signaling in basophils though its detailed mechanism is unclear [17].

Calcium signaling is critical for the activation of mast cells. In mast cells, tyrosine kinase Syk is recruited by aggregated FcεRI and phosphorylates phospholipase Cγ (PLCγ), leading to the generation of inositol 1, 4, 5-triphosphate (IP_3_). IP_3_ causes Ca^2+^ release from intracellular Ca^2+^ stores and activates Ca^2+^ influx via Ca^2+^ release-activated Ca^2+^ channels (CRAC) to replenish Ca^2+^ stores [22,23,24,25]. Therefore, it can be said that an increase in the intracellular Ca^2+^ is a necessary and sufficient stimulus to trigger degranulation in mast cells. We investigated whether the mechanism by which 5-HMF inhibits degranulation in mast cells involves the suppression of calcium signaling. We found that 5-HMF pretreatment inhibited FcεRI-mediated phosphorylation of PLC**γ**1 in DNP-HSA-stimulated BMMC. This observation suggests that 5-HMF suppresses calcium influx by modulating PLCγ phosphorylation and it agrees with previous reports that 5-HMF suppresses the elevation of intracellular Ca^2+^ concentration [17,26]. Thus, our study suggests that 5-HMF may prevent mast cell degranulation by regulating intracellular Ca^2+^ flux, but how 5-HMF might inhibit PLC**γ**1 phosphorylation is unclear and is a subject of further investigation.

We also showed that FcεRI-mediated cytokine production is inhibited by 5-HMF. When mast cells are activated via FcεRI, production of pro-inflammatory cytokines such as IL-6 occurs via the MAPK signaling pathway [27]. The main components of the MAPK signaling cascades include ERK1/2, SAPK/JNK, and p38 protein kinases, which are involved in number of physiological processes such as cell proliferation, differentiation, and inflammatory responses [28,29]. We observed that 5-HMF clearly inhibited FcεRI-mediated phosphorylation of ERK1/2, suggesting that 5-HMFcould disturb FcεRI-activated MAPK signaling pathway (Figure 6B). Collectively, our data provide further insights into the mechanism(s) controlling mast cell activation by 5-HMF that was previously unknown.

In conclusion, our findings suggest that the anti-allergic effect of Jabara is enhanced by fermentation, during which 5-HMF is produced. Further studies including in vivo experiments are required, but 5-HMF has potential to be useful for controlling allergy symptoms. We consider fermentation as a useful and effective way to produce 5-HMF as well as to improve the taste for consumption.

## 4. Materials and Methods

### 4.1. Fermentation of Jabara

Raw Jabara peel was aged in a fermenter for 4–12 weeks at a temperature of 70 °C and a humidity of 75% or higher with no additives or water added.

### 4.2. Reagents and Antibodies

Narirutin (141-09301) and 5-HMF (AC121460010) were purchased from FUJIFILM (Tokyo, Japan) and Thermo Fisher Scientific (Waltham, MA, USA), respectively. The following antibodies were commercially obtained: anti-phospho PLCγ1 (Tyr783), anti- PLCγ1 (Cell Signaling, Farmingdale, NY, USA), anti-phospho ERK1/2 (V803A), and anti-ERK1/2 (V114A) (Promega, Madison, WI, USA).

### 4.3. Mice

C57BL/6J and BALB/c mice were obtained from Japan SLC (Hamamatsu, Japan). The mice were maintained under specific pathogen-free conditions and were analyzed between 8 and 12 weeks of age for all studies performed. We obtained approval from the animal research committee at Suzuka University of Medical Science for all animal experiments performed (approved #75).

### 4.4. Cell Culture

Bone marrow-derived mast cells (BMMC) were prepared as described previously [30]. Briefly, 8-week-old C57BL6J mice were sacrificed and their bone marrow cells were cultured in RPMI 1640 supplemented with 10% heat-inactivated FBS, 10 mU/mL penicillin, 0.1 mg/mL streptomycin, and IL-3 in a 5% CO_2_ and 95% humidified atmosphere at 37 °C. After 4–5 weeks of culture, cell-surface expression of FcεRI and c-Kit was confirmed by FACSCalibur (BD Biosciences, San Diego, CA, USA).

### 4.5. BMMC Degranulation Assay

BMMC degranulation assay was performed as previously described [31]. Cells were sensitized with 0.5 µg/mL IgE for 12 h at 37 °C. After sensitization, the cells were washed twice with Tyrode’s buffer (10 mM HEPES pH 7.4, 130 mM NaCl, 5 mM KCl, 1.4 mM CaCl_2_, 1 mM MgCl_2_, and 5.6 mM glucose), and then suspended in the same buffer and stimulated with polyvalent dinitrophenyl-human serum albumin (DNP-HSA, Biosearch Technologies, Hoddesdon, UK) for 30 min at 37 °C. To measure β-hexosaminidase activity, 50 µL of supernatant or cell lysate was transferred in duplicates into a 96-well plate and 100 µL of 1.3 mg/mL p-nitrophenyl-N-acetyl-D-glucosaminide (in 0.1 M citrate, pH 4.5) was added to each well. The plate was then incubated for color development for 50 min at 37 °C. The enzyme reaction was stopped by adding 150 µL of 0.2 M glycine-NaOH (pH 10.2) followed by the measurement of absorbance at 405 nm. Tyrode’s buffer containing 1% Triton X-100 was used to lyse cells. The percentage of released β-hexosaminidase was calculated using the following formula:

Degranulation (%) = (OD supernatant) / (OD supernatant + OD lysate) × 100(1)

### 4.6. Measurement of Cytokines

Mast cells (0.5 × 10^5^ cells) were treated with Jabara extracts or 5-HMF for 6 h. IL-6 and IL-13 in the cell culture supernatants were measured with an ELISA kit (BD Biosciences, San Diego, CA, USA for IL-6; Thermo Fisher Scientific for IL-13).

### 4.7. Extraction from Fermented Jabara

Fermented of Jabara was pulverized and lyophilized for powder preparations. Before adding to the cultured mast cells, the entire fraction was dissolved in DMSO.

### 4.8. Identification of the Active Component from Fermented Jabara

The active fraction of fermented Jabara was subjected to Cosmosil 5C_18_-MS-II (φ4.6 mm × 150 mm, MeOH:H_2_O = 3:7) to yield two fractions (Fr. A and B). All fractions were tested for β- hexosaminidase release assay from mast cells. The chemical structure of the main active compound was identified based on HPLC-MS analysis and NMR analysis.

### 4.9. Cell lysates and Immunoblotting

Cell lysates and immunoblotting were performed as previously described [32]. BMMCs were harvested, lysed in lysis buffer (20 mM Tris-HCl pH 7.4, 150 mM NaCl, 1% NP-40, and proteinase inhibitor cocktail (Roche, Basel, Swizerland) for 30 min at 4 °C, and spun at 12,000× *g* at 4 °C for 30 min. The eluted and reduced samples were resolved by SDS-PAGE using a 5-20% gradient polyacrylamide gel (Wako Pure Chemical, Osaka Japan) and transferred to a PVDF membrane (Immobilon-P, Millipore, Billeria, MA, USA). For immunoblotting, the membranes were incubated with primary antibodies for 1 h at room temperature, and then incubated with HRP-conjugated anti-mouse (Thermo Fisher Scientific) or anti-rabbit (Thermo Fisher Scientific) antibody for 1 h at room temperature. After extensive washing of the membranes, immunoreactive proteins were visualized using the Western Lightning-ECL system (GE Healthcare Life Sciences, Buckinghamshire, England) according to the manufacturer’s recommendation. The PVDF membranes were exposed to Fuji RX film (Fujifilm). Densitometric analysis was performed using a LAS-4000 fluorescence image analyzer (Fujifilm, Tokyo, Japan).

### 4.10. Real-time PCR Analysis

Cells were homogenized with Sepasol RNAI (Nacalai Tesque, Kyoto, Japan), and total RNA was isolated following the manufacturer’s instructions. For standard RT-PCR, cDNA was synthesized from 1 μg of total RNA with reverse transcriptase (ReverTra Ace; TOYOBO, Osaka, Japan) and 500 ng of oligo (dT) primer (Life Technologies, Grand Island, NY, USA) for 30 min at 42 °C. A portion of the cDNA was used for real-time PCR. The relative expression of *Il-6* gene was determined compared to a reference gene *g3pdh* using the SYBR^®^ Green reagent (TaKaRa Bio inc., Kusatsu, Japan). Primers used in these experiments were purchased from TaKaRa, and the sequences were as follows: IL-6: forward primer, 5′- GAGGATACCACTCCCAACAGACC-3′ and reverse primer, 5′- AAGTGCATCATCGTTGTTCATACA-3′; G3PDH: forward primer, 5′- TTCACCACCATGGAGAAGGCCG-3′ and reverse primer, 5′- GGCATGGACTGTGGTCATGA-3′.

### 4.11. Cell Viability

BMMC was diluted to 1 × 10^6^ cells/mL in RPMI-1640 medium and 200 µL cell suspension was added to duplicate wells in a 96-well plate. 5-HMF was added to the final concentration of 200, 400 or 800 µg/mL in each well. Jabara extract was added to the final concentration of 400 µg/mL. The plate was incubated at 37 °C, and a 1:1 dilution of cell suspension and trypan blue (0.5%) was prepared at each timepoint. Cell viability was measured using an automated cell counter (TC20, Bio-Rad, Hercules, CA, USA).

### 4.12. Statistical Analysis

All data were statistically analyzed using Dunnett’s test or Student’s two-tailed t test with IBM SPSS Statistics software (version 24). Data were considered statistically significant when the *p* value was less than 0.05.

## Supplementary Materials

Supplementary materials can be found at https://www.mdpi.com/1422-0067/21/7/2472/s1.
