# Supplementary figures and images for: Identification of 5-Hydroxymethylfurfural (5-HMF) as an Active Component Citrus Jabara That Suppresses FcεRI-Mediated Mast Cell Activation
Source: Int J Mol Sci. 2020 Apr 2;21(7):2472. doi: 10.3390/ijms21072472 (PMC7177689; doi:10.3390/ijms21072472)

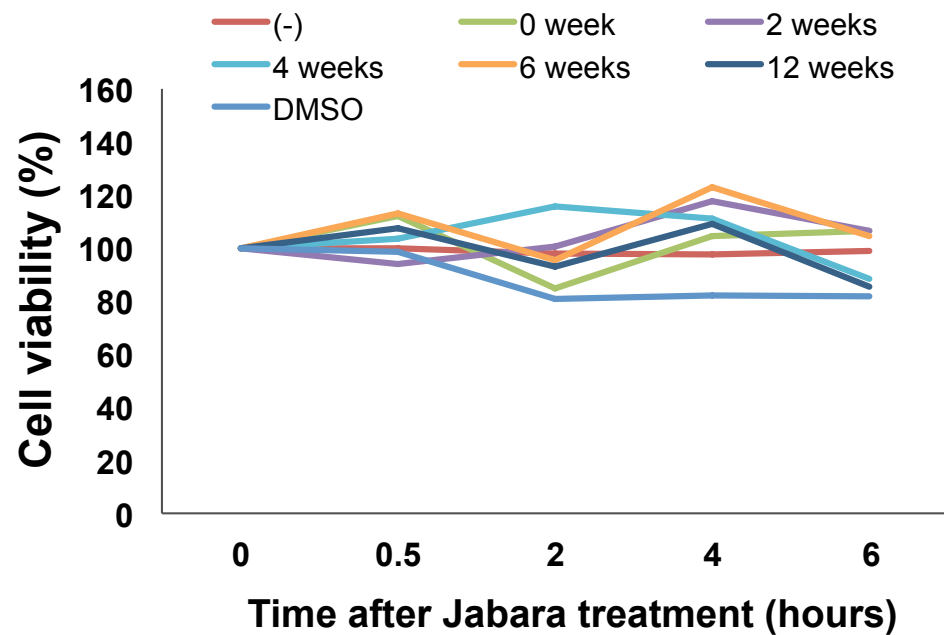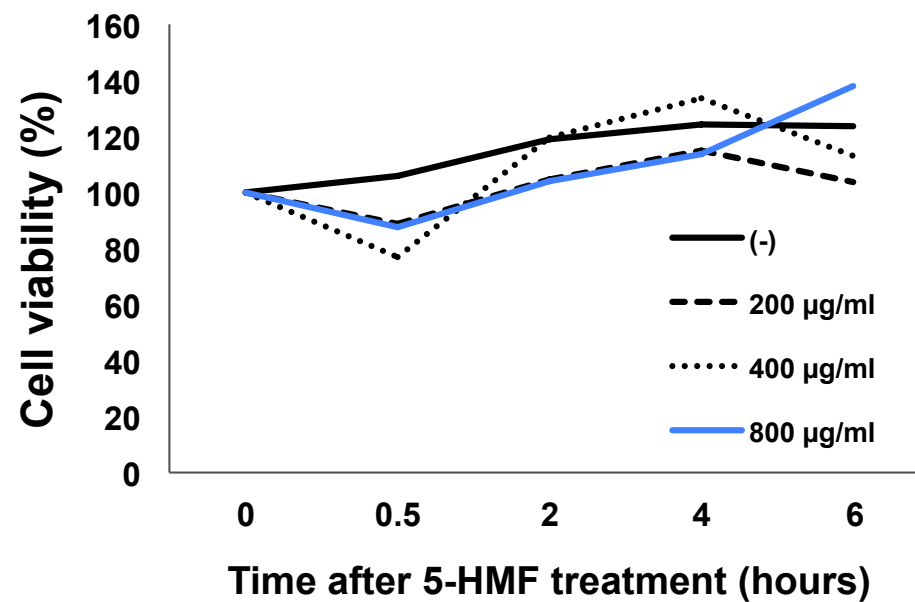

Supplement: Supplementary file 1 [file ijms-21-02472-s001.pdf]
